# Ricin Toxicity to Intestinal Cells Leads to Multiple Cell Death Pathways Mediated by Oxidative Stress

**DOI:** 10.3390/toxins17080400

**Published:** 2025-08-09

**Authors:** Francesco Biscotti, Massimo Bortolotti, Federica Falà, Antimo Di Maro, Andrea Bolognesi, Letizia Polito

**Affiliations:** 1 Department of Medical and Surgical Sciences—DIMEC, Alma Mater Studiorum, University of Bologna, Via San Giacomo 14, 40126 Bologna, Italy; francesco.biscotti2@unibo.it (F.B.); massimo.bortolotti2@unibo.it (M.B.); federica.fala@unibo.it (F.F.); 2 Department of Environmental, Biological and Pharmaceutical Sciences and Technologies (DiSTABiF), University of Campania ‘Luigi Vanvitelli’, Via Vivaldi 43, 81100 Caserta, Italy; antimo.dimaro@unicampania.it

**Keywords:** ricin, ribosome-inactivating proteins, toxic lectins, intestinal cells, oxidative stress, apoptosis, necroptosis

## Abstract

Ricin, a type 2 ribosome-inactivating protein, is a lethal toxin found in castor bean seeds. Although the systemic toxicity of ricin has been extensively studied, its localized effect on the gastrointestinal tract remains a critical concern, particularly in the case of oral ingestion. This study investigates the cytotoxic effects of ricin on human intestinal epithelial cell lines and its impact on epithelial barrier integrity. Ricin cytotoxicity was assessed on the intestinal-derived HT29 and Caco-2 cell lines using dose– and time–response assays, while the epithelial integrity was evaluated via Trans-Epithelial Electrical Resistance (TEER) measurements in Caco-2 monolayers. Cell death was determined through flow cytometry analysis, and the protective effects of cell death inhibitors and antioxidant scavengers were investigated on ricin-intoxicated cells. Ricin showed high cytotoxicity on HT29 and Caco-2 cells, with EC_50_ values in the nM range after 24–72 h of intoxication. Moreover, ricin strongly reduced TEER values in Caco-2 cells at 0.1–1 nM after 24 h of treatment. At a 1 nM concentration, ricin cytotoxicity can be significantly prevented by pre-incubating cells with the cell death inhibitors Z-VAD or necrostatin-1 and the antioxidant scavenger catalase, butylated hydroxyanisole or sodium pyruvate, demonstrating the involvement of apoptosis/necroptosis and oxidative stress in ricin cell death pathways and mechanisms.

## 1. Introduction

The castor bean plant (*Ricinus communis* L.) is a species of perennial flowering plant belonging to the Euphorbiaceae family. Castor bean toxicity mainly depends on its content of ricin, a highly potent ribosome-inactivating protein (RIP) [1]. Ricin belongs to the type II RIP family, characterized by a heterodimeric structure composed of an enzymatically active A-chain linked by a disulfide bond to a galactose-specific binding B-chain [2]. The A-chain has N-glycosylase activity and can catalytically depurinate several intracellular substrates, thus triggering multiple cell death pathways [3,4]. The B-chain, by binding to membrane glycoproteins and glycolipids, mediates the linkage of ricin to the cell surface, facilitating its endocytosis [5].

The acute toxicity of ricin is highly variable depending on the animal species and the route of exposure, as this determines the subsequent tissue distribution of the toxin. Initial symptoms may appear as early as 4–6 h after exposure, but serious symptoms may occur as late as 24 h after exposure. After oral ingestion, the gastrointestinal tract is severely compromised [6]. When seeds are ingested whole, they can pass through the gastrointestinal tract intact, whereas chewing facilitates the release of ricin. Unchewed seed portions may hide ricin, allowing its delayed release into the lower intestine, where it can cause great damage [7]. The most common symptoms of castor bean ingestion are vomiting, diarrhea and abdominal pain, but massive fluid and electrolyte loss has also been described, which can cause hypovolemic shock and multiorgan failure [8,9]. The lethal oral dose of ricin in humans is estimated to be between 1 and 20 mg/kg (about 5–10 seeds). This variability may depend on the degree of chewing, gastric content, age and comorbidities of the intoxicated subject [6,10]. For all these reasons, *Ricinus communis* has been included in the list of undesirable contaminants in the Annex to Directive 2002/32/EC [11] and by the Scientific Committee on Animal Nutrition of European Food Safety Agency [12].

On the other side, it should be considered that several parts of the castor bean plant, and the seed, have been employed since ancient times for medical purposes and are sometimes still used in some folk medicines [13]. The first written record of these utilizations in ancient Egypt can be found in the “Ebers Papyrus”, a medical treatise that dates back to approximately 1500 BC, where an entire section is devoted to castor beans [13]. Even then, castor bean toxicity was well known; in fact, only very small amounts of seeds are utilized to prepare drugs for oral ingestion. In Chinese traditional medicine, the seeds are used for anthelmintic activity [14]. In Ayurvedic medicine, castor bean is used in digestive disorders and as an anthelmintic, anti-bacterial and anti-diabetic agent [15,16]. In American folk medicine, castor bean leaf poultice is used for stomach aches [17]. In the Mediterranean area and Western countries, castor bean derivatives are used as an aperient and purgative [18].

For all the reasons outlined above, it is important to better elucidate the actual effect of ricin on intestinal cells. Although systemic toxicity of ricin has been extensively studied, its localized effects on the gastrointestinal tract remain a critical concern, particularly in cases of oral exposure. The intestinal epithelium serves as a primary barrier against ingested toxins and pathogens. Ricin’s cytotoxic impact on intestinal epithelial cells results in severe mucosal injury, increased epithelial permeability and inflammation. These effects compromise gut barrier function and can facilitate systemic dissemination of the toxin and other luminal pathogens or antigens.

The aim of this study is to investigate the cytotoxic effects of ricin on two human colon adenocarcinoma cell lines, HT29 and Caco-2, focusing on the effective dose, response time and cell death pathways. This study seeks to clarify the pathogenic mechanisms underlying cell death, with particular attention to the involvement of oxidative stress.

## 2. Results

### 2.1. Cytotoxic Effects of Ricin on Colon Adenocarcinoma Cells

**Figure 1:**
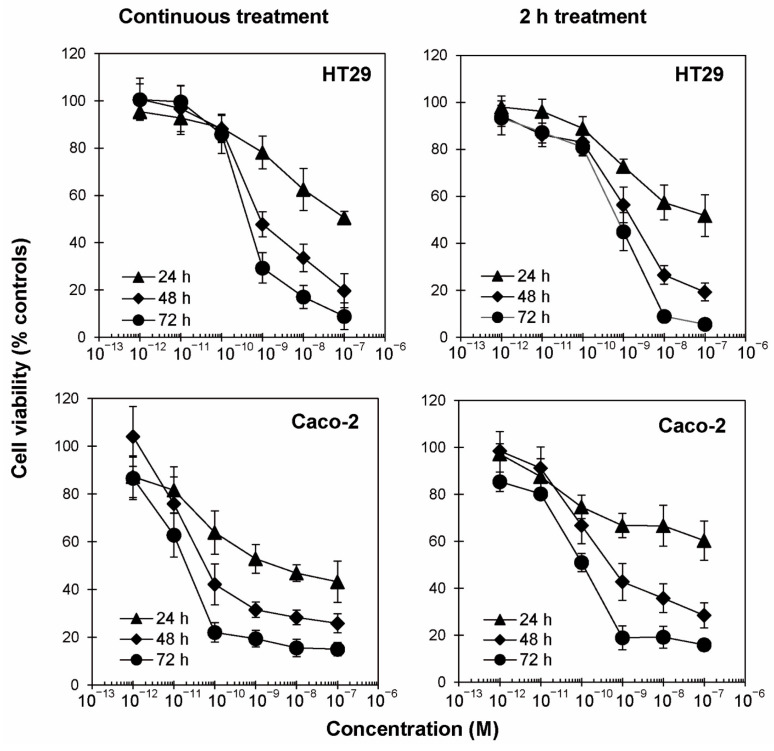
Concentration– and time–response experiments. HT29 and Caco-2 cells (3 × 10^3^/well) were treated with scalar concentrations of ricin for 24, 48 and 72 h in continuous treatment with ricin (**left** panels) or after a 2 h treatment with ricin followed by incubation for the same times (24, 48, 72 h) with medium alone (**right** panels). In both conditions, viability was evaluated using a colorimetric assay based on MTS reduction. Data are expressed as mean ± SD from three independent experiments, each carried out in triplicate. All results are plotted as relative percentage of untreated controls.

The cytotoxic effects of ricin were evaluated on two human colon adenocarcinoma cell lines, namely HT29 and Caco-2. Cells were treated with scalar concentrations of ricin (10^−12^–10^−7^ M). Cell viability experiments were carried out in two different conditions: (i) continuous incubation and (ii) 2 h incubation. In both conditions, the viability was evaluated after 24, 48 and 72 h (Figure 1).

While a viability-reducing effect was quite predictable on cells continuously exposed to ricin for long times (24–72 h), we had no idea what the effect of incubating cells with ricin for only 2 h might be. In continuous incubation experiments, ricin affected cell viability in a time- and dose-dependent manner (Figure 1). After 24 h, ricin significantly lessened cell viability with respect to untreated controls, already at concentrations of 10^−9^ and 10^−10^ M (*p* ≤ 0.0001), in HT29 and Caco-2 cells, respectively. A more marked decrease in cell viability was reported with longer incubation times. In HT29 cells, after 48 and 72 h, viability started to significantly decrease at 10^−9^ M (*p* ≤ 0.0001). Conversely, in Caco-2 cells, after 48 and 72 h, viability started to significantly decrease at 10^−10^ M (*p* ≤ 0.0001) or 10^−11^ M in the case of continuous incubation at 72 h (*p* ≤ 0.0001). After 48 h, viability was less than 20% at the maximum tested concentration (10^−7^ M). A greater reduction was evident after 72 h of incubation, mainly in Caco-2 cells, for which it was possible to reach a plateau of viability at less than 20% of controls with 10^−10^ M ricin.

Surprisingly, 2 h of incubation with ricin was sufficient to produce an effect on intestinal cell viability, detected after 24–72 h, quite similar to that reported with continuous incubation experiments (Figure 1).

Interestingly, the concentrations of ricin that reduce cell viability by 50% (EC_50_) calculated after 48 and 72 h from the curves resulting from the 2 h treatment experiments were very similar (with differences lower than 1 logarithm) to those calculated from the continuous incubation experiments (Table 1). Thus, the effect of ricin, evaluated after a total time of 72 h, remains substantially unchanged regardless of the contact time of ricin with the cells (2 h or continuous treatment). This suggests that 2 h contact with the toxin is sufficient to induce the almost full cytotoxic effect.

### 2.2. Effects of Ricin on Barrier Integrity in Caco-2 Monoculture and Caco-2/HT29 Co-Culture Cell Models

The measurement of Trans-Epithelial Electrical Resistance (TEER) is a widely used approach for assessing the growth and differentiation of Caco-2 epithelial monolayers in vitro. This quantitative, non-invasive and non-destructive technique enables the real-time evaluation of tight junction formation in living cells. To compare the extent of ricin intoxication effects on intestinal cells, both continuous and 2 h treatments were assessed. The use of the Caco-2/HT29 co-culture model is justified by the need to more accurately replicate the heterogeneous composition of the intestinal epithelium and the interaction of its cell types that cannot be fully represented by Caco-2 monoculture alone [19].

**Figure 2:**
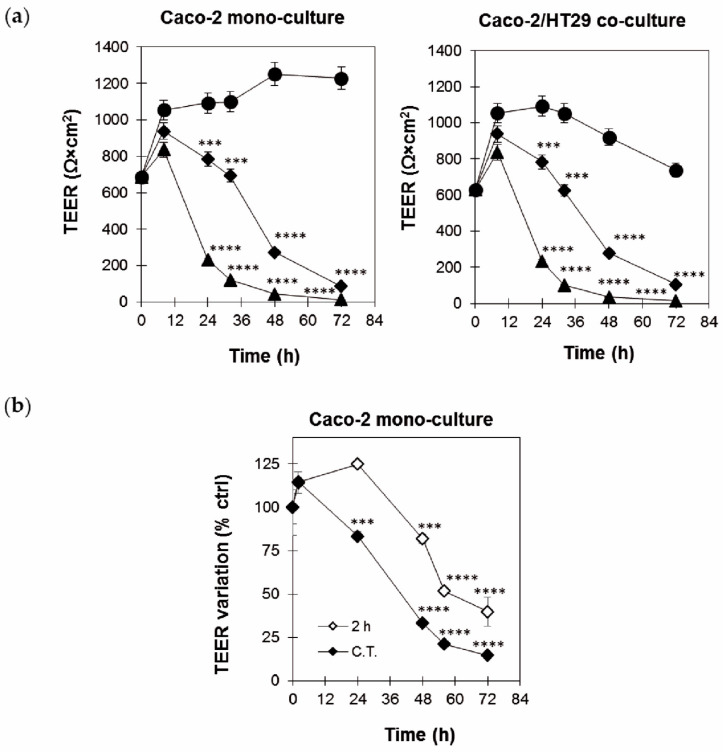
Dose- and time-dependent TEER changes across Caco-2 and Caco-2/HT29 monolayers. Cell monolayers were prepared by seeding 6.25 × 10^4^ cells on Transwell inserts and maintained for about 10 days, replacing culture medium every 2–3 days. Caco-2 and Caco-2/HT29 monolayers were used when initial TEER values were ≥500 Ω × cm^2^ (about 10 days). (**a**) Cell monolayers were treated with medium without ricin (●) or ricin at concentrations of 10^−9^ (▲) and 10^−10^ M (◆). TEER values were assessed at 0, 8, 24, 32, 48 and 72 h after ricin intoxication. (**b**) Caco-2 monolayers were treated for 0, 2, 24, 48, 56 and 72 h with 10^−10^ M ricin either continuously (C.T.) (◆) or with ricin for 2 h followed by incubation with medium alone (2 h) (◊). The results are reported as a percentage of untreated controls. Results are expressed as mean ± SD of three independent experiments with three replicates in each experiment. ANOVA/Bonferroni, with Dunnett’s test (*** *p* ≤ 0.001; **** *p* ≤ 0.0001 versus untreated controls).

In continuous experiments, ricin reduced TEER values in both the Caco-2 monoculture and Caco-2/HT29 co-culture cell models. At 10^−9^ M concentration, ricin reduced TEER after 24 h, showing TEER values 5-fold lower than those of controls. At 10^−10^ M concentration, ricin was also able to reduce TEER values by about 1.3-fold with respect to control cells (Figure 2a).

According to the results observed in cell viability experiments, a 2 h exposure to ricin was sufficient to compromise the integrity of Caco-2 monolayers, starting from 48 to 72 h. TEER was not affected by ricin at 24 h, while a marked decrease was observed between 24 and 48 h. Comparing 2 h to continuous treatment, we can observe that TEER variation was less pronounced at both 24 and 48 h in the 2 h treatment, showing approximately 1.5-fold and 2.5-fold lower variation, respectively (Figure 2b).

### 2.3. Evaluation of Cell Death Induced by Ricin in HT29 and Caco-2 Cells

**Figure 3:**
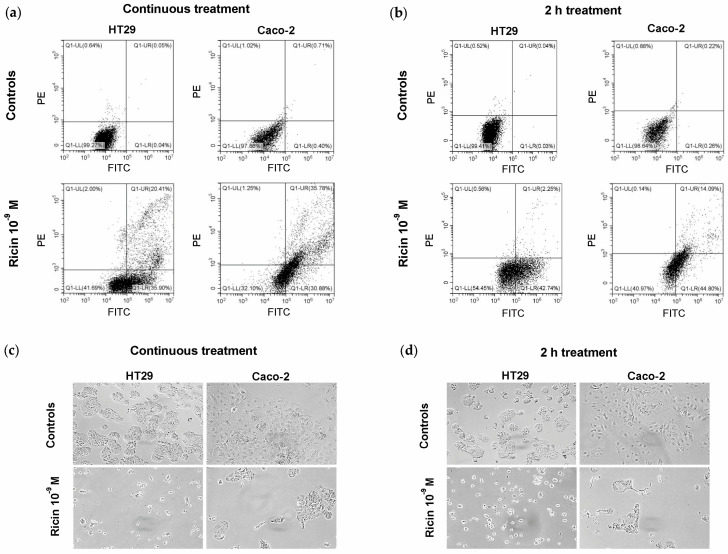
Evaluation of cell death induced by ricin in HT29 and Caco-2 cells through morphological analysis and flow cytometry analysis after Annexin V-EGFP/PI staining. HT29 and Caco-2 cells were treated with 10^−9^ M ricin (**a**) in continuous treatment for 48 h or (**b**) in 2 h treatment followed by incubation with complete medium alone for 48 h. Plots show live cells (**lower-left** quadrant; PI-negative and EGFP-negative), necrotic cells (**upper-left** quadrant; PI-positive and EGFP-negative), late apoptotic and necroptotic cells (**upper-right** quadrant; PI-positive and EGFP-positive) and early apoptotic cells (**lower-right** quadrant, PI-negative and EGFP-positive). The plots are representative of three independent experiments. Morphology of intestinal cells after continuous treatment (**c**) and 2 h treatment (**d**) was assessed using phase-contrast microscopy (magnification 100×).

The cell death pathways activated in ricin-treated cells were evaluated by double staining with Annexin V-Enhanced Green Fluorescent Protein (EGFP)/Propidium iodide (PI) through flow cytometry analysis. After 48 h of continuous treatment with ricin at 10^−9^ M concentration, 56.31% of HT29 cells and 66.66% of Caco-2 cells were in early/late apoptosis. However, we can hypothesize that a portion of the cells in late apoptosis may represent necroptotic cells, as reported in [20]. No significative involvement of necrosis was detected after ricin intoxication (Figure 3a, Table 2). Similar results were obtained at 48 h after a 2 h treatment with 10^−9^ M ricin. In this case, the percentage of cells in early apoptosis is higher than that of cells in late apoptosis/necroptosis. Anyway, in this treatment condition, no involvement of necrosis was observed (Figure 3b, Table 2).

The morphological changes in ricin-treated intestinal cells, after continuous treatment (Figure 3c) and after 2 h treatment (Figure 3d), were evaluated using phase-contrast microscopy. Cells showed morphological features characteristic of apoptosis, such as cell shrinkage and cytoplasmic condensation, thus confirming results obtained via cytofluorimetric analysis.

### 2.4. Effects of Cell Death Inhibitors on Ricin Cytotoxicity in HT29 and Caco-2 Cells

Flow cytometry experiments showed the involvement of apoptosis and necroptosis, both in continuous treatment and 2 h treatment with ricin. In order to evaluate the role of apoptosis and necroptosis in the pathogenesis of intoxication induced by ricin in intestinal cells, cell viability experiments were conducted, pre-treating cells for 3 h with the pan-caspase inhibitor Z-VAD and with the necroptosis inhibitor necrostatin-1 (NEC). Afterwards, the cells were treated for 2 h with ricin at 10^−9^ M, and the cytotoxicity was evaluated after 48 and 72 h.

**Figure 4:**
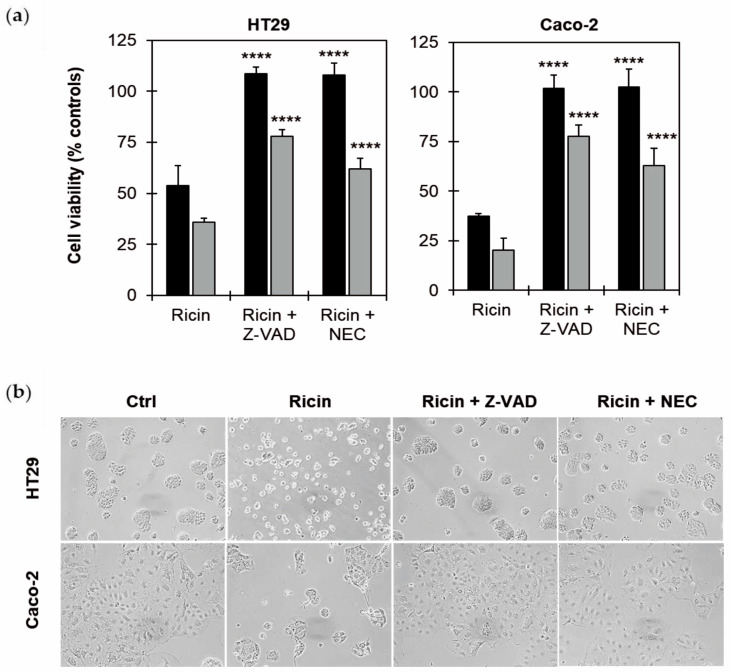
Effect of cell death inhibitors on ricin cytotoxicity. (**a**) Viability of HT29 and Caco-2 cells (3 × 10^3^/well), treated with 10^−9^ M ricin, without or in presence of 100 μM Z-VAD or 100 μM necrostatin-1 (NEC). Cells were pre-incubated with the inhibitors 3 h prior ricin treatment. Cells were then treated for 2 h with ricin and incubated for 48 h (black columns) and 72 h (gray columns) in complete medium alone. Histograms report means ± S.D. of three independent experiments, each conducted in triplicate. Data were analyzed via the Mann–Whitney U test. Asterisks indicate the significant difference in each experimental condition between ricin alone and ricin plus inhibitors (**** *p* ≤ 0.0001). (**b**) Morphological analysis of cells treated with ricin in presence or in absence of cell death inhibitors was assessed after 48 h through phase-contrast microscope (100× magnification).

As shown in Figure 4a, the cell death inhibitors were able to significantly (*p* ≤ 0.0001) increase the survival of ricin-treated HT29 and Caco-2 cells, giving total protection against ricin intoxication at 48 h. These results were confirmed by morphological analysis, showing that the cells, pre-treated with Z-VAD and NEC, maintained the morphological features of untreated cells (Figure 4b). The inhibitors significantly protected cells from ricin-induced damage, even after 72 h of incubation, although the protection was not complete, about 75% with Z-VAD and 65% with NEC. These data clearly demonstrate the simultaneous activation of both caspase-dependent apoptosis and necroptosis in ricin-treated intestinal cells.

### 2.5. Effects of ROS Scavengers on Ricin Cytotoxicity in HT29 and Caco-2 Cells

The involvement of oxidative stress was evaluated through cell viability experiments, pre-treating cells for 3 h with three different reactive oxygen species (ROS) scavengers, catalase (CAT), sodium pyruvate (NaPyr) and butylated hydroxyanisole (BHA). The cells were further treated for 2 h with ricin at 10^−9^ M, and the cytotoxicity was evaluated after 48 and 72 h.

**Figure 5:**
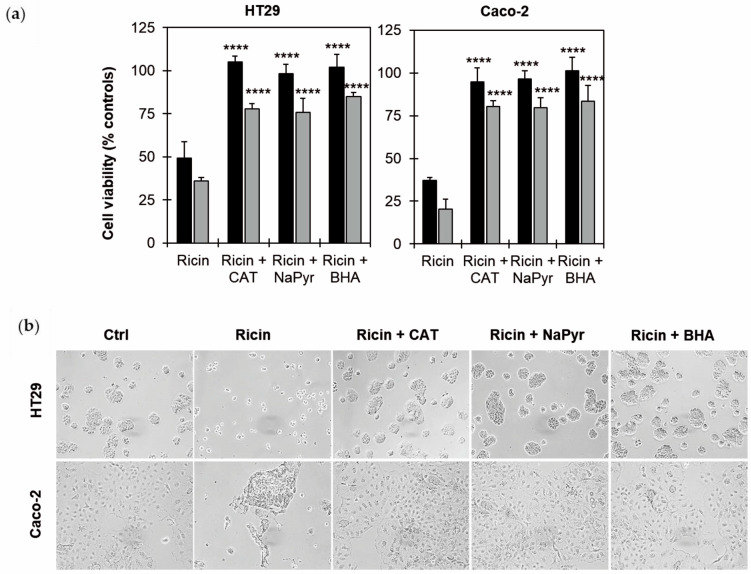
Effect of ROS scavengers on ricin cytotoxicity. (**a**) Viability of HT29 and Caco-2 cells (3 × 10^3^/well), treated with 10^−9^ M ricin, without or in presence of 10 U/mL catalase (CAT), 1 mM sodium pyruvate (NaPyr) or 30 μM BHA. Cells were pre-incubated with the scavengers 3 h prior to ricin treatment. Cells were then treated for 2 h with ricin and incubated for 48 h (black columns) and 72 h (gray columns) in complete medium alone. The results are expressed as means ± S.D. of three independent experiments, each conducted in triplicate. Data were analyzed via the Mann–Whitney U test. Asterisks indicate the significant difference in each experimental condition between ricin alone plus scavengers (**** *p* ≤ 0.0001). (**b**) Morphological analysis of cells treated with ricin in presence or in absence of scavengers was assessed after 48 h through phase-contrast microscope (100× magnification).

As shown in Figure 5a, all the tested scavengers gave significant protection from ricin’s toxic effects (*p* ≤ 0.0001), resulting in cell viability values at 48 and 72 h of approximately 100% and 80%, respectively, in both cell lines. These results were confirmed by morphological analysis, showing that the treated cells, if pre-treated with scavengers, had morphological features similar to those of untreated cells (Figure 5b).

## 3. Discussion

This study aimed to clarify some key aspects of ricin-induced cytotoxicity in two human colon adenocarcinoma cell lines, namely HT29 and Caco-2, focusing on the triggered cell death pathways and the possible involvement of oxidative stress.

HT29 cells are receiving special interest in studies focused on food digestion and bioavailability due to the ability to express the characteristics of mature intestinal cells [21]. Caco-2 cells are widely recognized for their ability to spontaneously differentiate into enterocyte-like cells, forming a polarized monolayer with tight junctions, making them an excellent model for studying intestinal barrier function and drug absorption [22,23]. Knowledge of damage mechanisms induced by ricin on intestinal cells is crucial to better understand the passage of the toxin from the intestine to the bloodstream and possibly to develop countermeasures against oral poisoning by ricin or castor beans.

Consistent with previous research highlighting the significant impact of ricin on the gastrointestinal tract [6,24,25,26,27], our results demonstrate a dose- and time-dependent viability reduction in both HT29 and Caco-2 cells after ricin exposure. Due to the characteristics of intestinal transit, prolonged contact between ricin and enterocytes in vivo is not possible. So, to better simulate the intestinal condition, we decided to expose cells for a limited time (2 h) to ricin, comparing its effect to that of continuous incubation. Interestingly, evaluating the EC_50_ values obtained from the cytotoxicity curves, continuous or 2 h treatment with ricin resulted in a very similar cytotoxic effect. In Caco-2 cells, the difference between the two types of incubation was slightly more marked than in HT29 cells. These results point out that the toxic effect of ricin on intestinal cells does not require contact time greater than 2 h. Evidently, ricin binding and endocytosis to intestinal cells trigger a cascade of events that result in long-term cellular damage, regardless of prolonged toxin presence. This finding is particularly relevant when considering how ricin is ingested in real-world scenarios. In these cases, the contact time between the toxin and the intestinal epithelium is always transitory and depends on various factors, including whether seeds or purified ricin have been ingested, whether the seeds have been ingested whole or chewed, the presence or absence of castor oil and the stomach contents. Furthermore, different individual factors of the patients, mainly the age and health of the subject, are also responsible for the progress and the symptomatology [28].

The effect of ricin on the intestinal barrier integrity was determined by detecting TEER both on Caco-2 monoculture and also on Caco-2/HT29 co-culture, the latter to better simulate the heterogeneity of the intestinal microenvironment and to allow for a more accurate assessment of the permeability of the toxin [19]. TEER experiments confirmed the cytotoxicity results, demonstrating a dose- and time-dependent reduction in barrier integrity after ricin intoxication, in both Caco-2 monocultures and Caco-2/HT29 co-culture models. In the 2 h treatment experiments, the decline in TEER was delayed in comparison to continuous treatment, but it was still clearly evident. Further, in this case, 2 h exposure to the toxin was sufficient to irreversibly compromise cell monolayer integrity. Impairment of the epithelial barrier is critical, as it can lead to increased permeability, facilitating the systemic dissemination of the toxin and of other luminal pathogens, thus exacerbating the overall pathology [27].

Some evidence has been reported about inflammation and oxidative stress involvement in different models of ricin intoxication. In addition to protein synthesis blockage, ricin and other RIPs were described to cause the activation of intracellular stress pathways, inflammation and cell death via apoptosis, necroptosis or necrosis [29,30,31,32,33,34]. However, very little information is available about intestinal cell intoxication. Understanding ricin’s specific interactions with intestinal cells is crucial for developing effective countermeasures against ricin or castor bean oral intoxication and for improving gastrointestinal models in toxicological research.

Our experiments on cell death mechanisms revealed a predominant role for apoptosis in ricin-intoxicated HT29 and Caco-2 cells, with a significant percentage of cells undergoing apoptosis after 48 h of ricin exposure without necrosis involvement. It is important to emphasize that flow cytometry analysis allowed us to hypothesize the involvement of necroptosis alongside apoptosis [20]. This was indeed confirmed by subsequent experiments with inhibitors of apoptosis and necroptosis. These experiments demonstrated the involvement of necroptosis in the ricin-induced cell death, in a way perhaps not qualitatively but certainly quantitatively similar to apoptosis. The protection afforded by pan-caspase inhibitor Z-VAD and by necrostatin-1 (NEC) was complete in both cases at 48 h, whilst at 72 h Z-VAD provided slightly greater protection than NEC.

In the present study, the involvement of necroptosis in intestinal cells intoxicated by ricin is described for the first time. Necroptosis, unlike apoptosis, is a caspase-independent form of programmed cell death, characterized by cellular swelling and plasma membrane rupture, leading to an inflammatory response [35,36]. Necroptosis can play an important role in chronic inflammatory conditions of the gut, promoting inflammatory bowel disease and impairing intestinal barrier integrity [37].

The finding that oxidative stress plays a key role in ricin-mediated intestinal cell damage is a major advance in this research, and this appears to agree with some evidence obtained in vivo in mouse hepatic and renal cells [38]. Reactive oxygen species (ROS) are essential for cellular signaling and response to stress. The level of ROS and the type of ROS determine the ability of cells to undergo cell death. ROS play essential roles in all forms of cell death, and they also control and determine the type of cell death that occurs in any given cell. Indeed, ROS may act as a rheostat, allowing different cell death mechanisms to be engaged and crosstalk with different cell death types. In general, limited mitochondrial damage leads to activation of the apoptotic intrinsic pathway, whereas widespread mitochondrial damage leads to high levels of ROS and lipid oxidation, causing plasma membrane damage and necroptosis. Furthermore, cell sensitivity to ROS also depends on the integrity of the intracellular antioxidant pathways [39]. In our experimental models, pre-treatment with ROS scavengers completely prevented ricin’s toxic effects at 48 h and significantly protected cells after 72 h, preserving the normal cell morphology. This strongly suggests that ricin induces the generation of ROS, which in turn contribute significantly to the observed cytotoxicity and cell death of intestinal cells.

BHA is a widely used antioxidant and preservative in food that minimally affects the human gut microbiome. For most food, the legal limit for BHA is 0.01% (corresponding to about 0.5 mM) of the oil or fat content of the food [40]. In previous in vivo studies on the mouse small intestine, BHA showed chemo-preventive properties, attributed to its ability to activate the transcription factor NF-E2 p45-related factor 2, which directs central genetic programs of detoxification and protection against oxidative stress [41,42]. Notably, in our experiments, a dose of BHA lesser (i.e., 30 µM) than that commonly used in food was able to protect intestinal cells from ricin toxicity.

Our results provide indirect evidence of oxidative stress involvement in ricin-induced intestinal cell death mechanisms, supporting the idea that antioxidants can protect intestinal cells from ricin-induced toxicity. Several further studies could be scheduled in vitro and vivo to confirm the results obtained in our experiments. Our studies pave the way for further experiments aimed to design strategies against ricin oral intoxication. The ultimate goal of this type of study is certainly the development of a therapeutic strategy against oral ricin intoxication, but much more research and global efforts are still needed.

## 4. Conclusions

In conclusion, our findings show that ricin induces significant cytotoxicity in intestinal cells through a complex interplay of apoptotic/necroptotic pathways regulated by oxidative stress. We detected a rapid onset of toxicity and significant protection by both cell death inhibitors and ROS scavengers. Overall, these results contribute to a deeper understanding of ricin’s specific interactions with intestinal cells and reinforce the potential therapeutic strategies for targeting oxidative stress and programmed cell death pathways in developing effective countermeasures against oral ricin poisoning.

## 5. Materials and Methods

### 5.1. Ricin Purification

The type 2 RIP ricin was purified from the seeds of *Ricinus communis,* as previously described [43], with some modifications. Briefly, castor bean extract was purified via affinity chromatography on acid-treated Sepharose CL-6B matrix (GE Healthcare, Buckinghamshire, UK) and eluted stepwise with 0.2 M galactose in buffer containing 0.14 M NaCl, 5 mM sodium phosphate buffer, pH 7 (PBS), as described in [33].

### 5.2. Cell Maintenance

The human colon adenocarcinoma cell lines HT29 (lot number 300215-921) and Caco-2 (lot number 300137-220424) were from Cytion (Eppelheim, Germany). Cells were cultured in complete Eagle’s minimum essential medium, supplemented with 2 mM L-glutamine, 10% (*v*/*v*) heat-inactivated fetal bovine serum, 1% (*w*/*v*) non-essential amino acids, 100 U/mL penicillin G and 100 μg/mL streptomycin (Sigma Aldrich, Saint Louis, Dorset, MO, USA). Cells were maintained at 37 °C in a humidified atmosphere of 5% CO_2_ in a HeraCell Heraeus incubator (Hanau, Germany), according to manufacturer instructions. Cell lines were routinely sub-cultured at 80–90% confluence, and the absence of mycoplasma infection was routinely checked in all the tested cell lines.

### 5.3. Cell Viability

Cell viability was determined, as already described in [33]. Briefly, HT29 and Caco-2 cells (3 × 10^3^/well) were seeded in 96-well microtiter plates (Falcon, Mexico City, Mexico). After 24 h, cells were incubated with scalar concentrations of ricin (from 10^−12^ to 10^−7^ M) in two different conditions: (i) continuous incubation and (ii) 2 h treatment with ricin. In both conditions, cell viability was evaluated after 24, 48 and 72 h. At the end of the incubation times, the medium was replaced with 100 μL/well of fresh medium containing 20 μL/well of CellTiter reagent (Promega Corporation, Madison, WI, USA). After 2 h incubation at 37 °C, the absorbance at 492 nm was measured.

### 5.4. Measurement of Trans-Epithelial Electrical Resistance (TEER)

Trans-Epithelial Electrical Resistance (TEER) measurement is widely used to assess the integrity and permeability of cell-based models. The TEER was evaluated on both Caco-2 cell monoculture and Caco-2/HT29 co-culture cell models. Cells (6.25 × 10^4^ Caco-2 cells/cm^2^; 6.25 × 10^4^ Caco-2/HT29 cells in a 9:1 ratio) were seeded in 24-well Transwell inserts (Falcon) with 0.4 µm transparent polyester membrane. When TEER reached values ≥ 500 Ω × cm^2^ (about 10 days), cells were treated with ricin at 10^−9^ and 10^−10^ M, and TEER was measured after 0, 8, 24, 36, 48 and 72 h. For 2 h treatment experiments, Caco-2 monolayers were subjected to a 2 h treatment of ricin at a concentration of 10^−10^ M, followed by incubation with complete medium alone. TEER measurements were assessed at baseline (0 h), at the end of the 2 h treatment and during the other incubation times (24, 48, 56 and 72 h).

All experiments were carried out using a digital voltohmmeter Millicel^®^ ERS 3.0 (Merck, Rahway, NJ, USA). A Transwell insert with no cells served as a blank control. TEER values (Ω × cm^2^) from monoculture cell models were calculated using Equation (1):

TEER = (R − R_blanck_) × A (1)

where R is the resistance measured across the cell layer(s), R_blank_ is the resistance of a blank Transwell insert and A is the surface area of a Transwell insert.

### 5.5. Evaluation of Cell Death Mechanisms Involved Through Flow Cytometry Analysis

The involvement of apoptosis, necroptosis and necrosis was evaluated through flow cytometry analysis using the Annexin V-EGFP/PI, as already described in [33]. Briefly, cells (4 × 10^5^) were incubated, continuously or only for 2 h, with ricin 10^−9^ M for a total time of 48 h. After this incubation time, cells were collected, pelleted, washed twice in cold PBS and resuspended in 300 μL of binding buffer, containing Annexin V-EGFP (3 μL) and PI (3 μL). After 10 min incubation in the dark at room temperature, cells were analyzed via flow cytometry CytoFLEX S (Beckman Coulter, Brea, CA, USA), using the CytExpert Software (version 2.4.0.28, 2011–2019). Necrotic cells were PI-positive and EGFP-negative; late apoptotic and necroptotic cells were PI-positive and EGFP-positive; early apoptotic cells were PI-negative and EGFP-positive.

### 5.6. Evaluation of the Involvement of Apoptosis/Necroptosis and Oxidative Stress Using Cell Death Inhibitors and ROS Scavengers

Ricin cytotoxicity was evaluated on HT29 and Caco-2 cells (3 × 10^3^/well in 96-well microtiter plates) pre-treated with 100 μM cell death inhibitors, Z-VAD or necrostatin-1 (NEC) and with the ROS scavengers, 10 U/mL catalase (CAT), 1 mM sodium pyruvate (NaPyr) or 30 μM butylated hydroxyanisole (BHA). The concentrations of the cell death inhibitors and ROS scavengers are the highest concentrations being not toxic for HT29 and Caco-2 cells in preliminary tests. Cell death inhibitors and ROS scavengers were added to cells 3 h before ricin treatment. Subsequently, both inhibitors and scavengers were removed, and the cells were treated for 2 h with 10^−9^ M ricin. After one wash with 100 μL/well PBS, cells were further incubated for 48 and 72 h in complete medium. Cell viability was evaluated as described above.

### 5.7. Morphological Analysis of Ricin-Treated Intestinal Cells

The morphological analysis of intestinal cells was performed in 6-well (for cytofluorimetric experiments) or in 96-well plates (for viability experiments) using a phase-contrast microscope with a digital camera (Nikon, Tokyo, Japan). Images were captured by X-Elit software, version 18.01.02 (Alexasoft, Florence, Italy).

### 5.8. Statistical Analysis

Statistical analyses were conducted using XLSTAT-Pro Software, version 6.1.9, 2003 (Addisoft, Inc., Brooklyn, NY, USA). The results are presented as means ± S.D. of three different experiments. The data were analyzed using ANOVA/Bonferroni test or Mann–Whitney U test. Dunnett’s test was used in addition to ANOVA, when necessary.

## Figures and Tables

**Table 1:** Concentrations of ricin that reduce cell viability by 50% (EC_50_), calculated through linear regression analysis of concentration–response curves. Concentration–response experiments were carried out in continuous treatment (C.T.) and in a 2 h treatment (2 h) with scalar concentrations of ricin.

	EC_50_ (M)			
Cell Line	Treatment	24 h	48 h	72 h
HT29	C.T.	6.96 × 10^−8^	8.82 × 10^−10^	4.31 × 10^−10^
	2 h	>10^−7^	1.52 × 10^−9^	7.22 × 10^−10^
Caco-2	C.T.	3.30 × 10^−9^	6.14 × 10^−11^	1.80 × 10^−11^
	2 h	>10^−7^	4.99 × 10^−10^	9.99 × 10^−11^

**Table 2:** Relative percentages of live, early apoptotic, late apoptotic/necroptotic and necrotic cells post-ricin treatment at 48 h with continuous treatment (C.T.) or 2 h treatment (2 h).

Cell Line	Treatment	Live	Early Apoptotic	Late Apoptotic/ Necroptotic	Necrotic
		Relative Percentage of Ricin-Treated Cells			
HT29	C.T.	41.69	35.90	20.41	2.00
	2 h	54.45	42.74	2.25	0.56
Caco-2	C.T.	32.10	30.88	35.78	1.25
	2 h	40.97	44.80	14.09	0.14

## Data Availability

The original contributions presented in this study are included in the article. Further inquiries can be directed to the corresponding authors.

## Abbreviations

The following abbreviations are used in this manuscript:

BHA	Butylated hydroxyanisole
C.T.	Continuous treatment
CAT	Catalase
EC_50_	Effective Concentration reducing cell viability by 50%
EGFP	Enhanced Green Fluorescent Protein
NaPyr	Sodium Pyruvate
NEC	Necrostatin-1
PBS	Sodium Phosphate Buffer
PI	Propidium Iodide
RIP	Ribosome-Inactivating Protein
ROS	Reactive Oxygen Species
TEER	Trans-Epithelial Electrical Resistance
